# A unique single nucleotide polymorphism in Agouti Signalling Protein (*ASIP*) gene changes coat colour of Sri Lankan leopard (*Panthera pardus kotiya*) to dark black

**DOI:** 10.1371/journal.pone.0269967

**Published:** 2023-07-13

**Authors:** M. G. C. Sooriyabandara, A. U. Bandaranayake, H. A. B. M. Hathurusinghe, S. M. Jayasundara, M. S. R. R. P. Marasinghe, G. A. T. Prasad, V. P. M. K. Abeywardana, M. A. Pinidiya, R. M. R. Nilanthi, P. C. G. Bandaranayake

**Affiliations:** 1 Department of Wildlife Conservation, Battaramulla, Sri Lanka; 2 Department of Computer Engineering, Faculty of Engineering, University of Peradeniya, Peradeniya, Sri Lanka; 3 Agricultural Biotechnology Centre, Faculty of Agriculture, University of Peradeniya, Peradeniya, Sri Lanka; Smithsonian Conservation Biology Institute, UNITED STATES

## Abstract

The Sri Lankan leopard (*Panthera pardus kotiya*) is an endangered subspecies restricted to isolated and fragmented populations in Sri Lanka. Among them, melanistic leopards have been recorded on a few occasions. Literature suggests the evolution of melanism several times in the Felidae family, with three species having distinct mutations. Nevertheless, the mutations or other variations in the remaining species, including Sri Lankan melanistic leopard, are unknown. We used reference-based assembled nuclear genomes of Sri Lankan wild type and melanistic leopards and *de novo* assembled mitogenomes of the same to investigate the genetic basis, adaptive significance, and evolutionary history of the Sri Lankan melanistic leopard. Interestingly, we identified a single nucleotide polymorphism in exon-4 Sri Lankan melanistic leopard, which may completely ablate Agouti Signalling Protein (*ASIP*) function. The wild type leopards in Sri Lanka did not carry this mutation, suggesting the cause for the occurrence of melanistic leopords in the population. Comparative analysis of existing genomic data in the literature suggests it as a *P*. *p*. *kotiya* specific mutation and a novel mutation in the *ASIP*-gene of the Felidae family, contributing to naturally occurring colour polymorphism. Our data suggested the coalescence time of Sri Lankan leopards at ~0.5 million years, sisters to the *Panthera pardus* lineage. The genetic diversity was low in Sri Lankan leopards. Further, the *P*. *p*. *kotiya* melanistic leopard is a different morphotype of the *P*. *p*. *kotiya* wildtype leopard resulting from the mutation in the *ASIP*-gene. The ability of black leopards to camouflage, along with the likelihood of recurrence and transfer to future generations, suggests that this rare mutation could be environment-adaptable.

## Introduction

**Fig 1:**
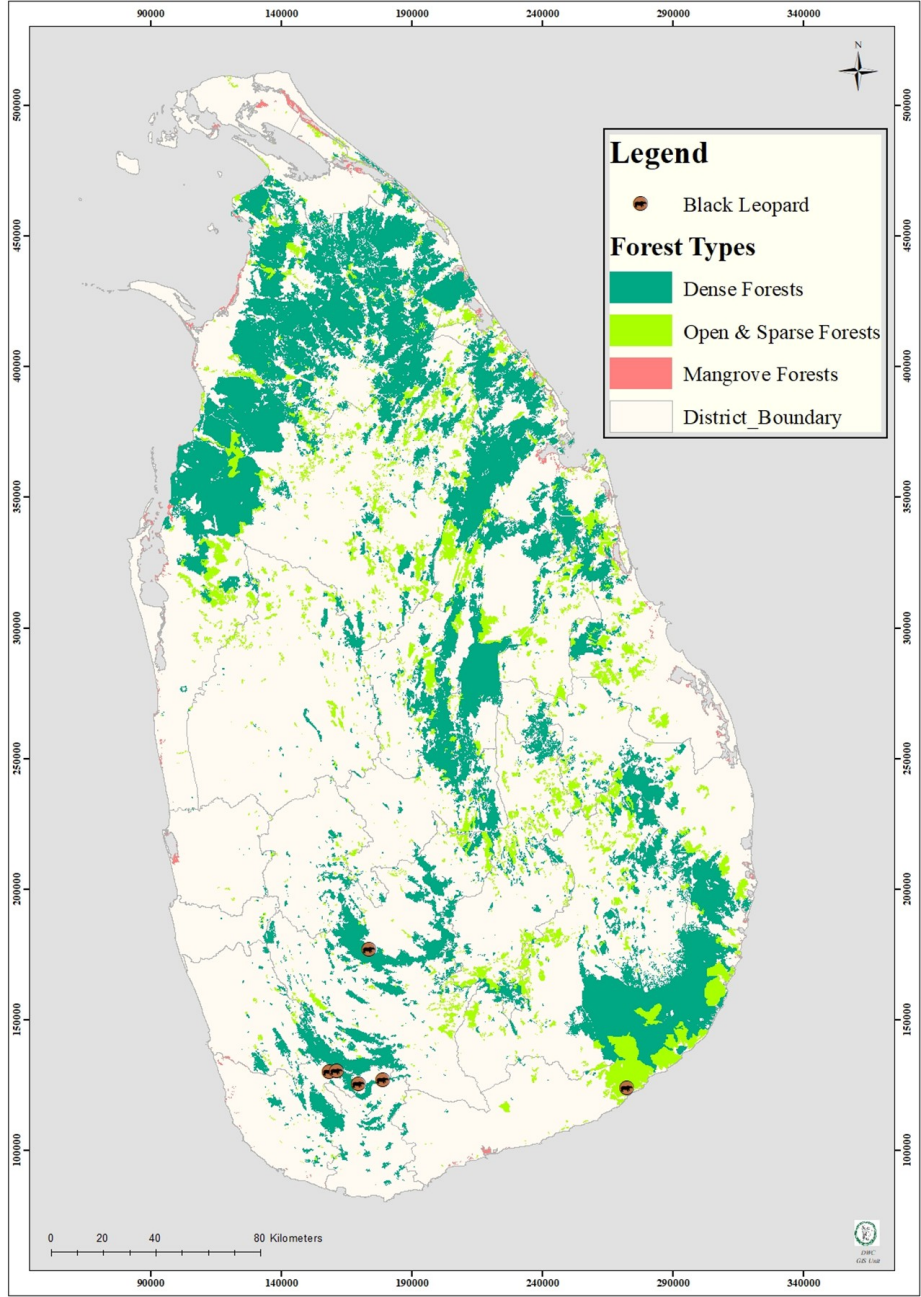
The geographic distribution and habitat association of melanistic leopard morphotypes in Sri Lanka.

Leopards are critically endangered in many habitats [1], including Sri Lanka [2]. *Panthera pardus kotiya*, the Sri Lankan leopard, is one of nine unique sub-species and the second remaining island leopard in the world [1, 3]. Leopard subspecies are characterized using genetic, morphological, and geographical information [4]. Based on previous DNA analysis [5], the nine subspecies recognized in 1996 are; *Panthera pardus pardus* (Linnaeus, 1758): Africa, *Panthera pardus nimr* (Hemprich & Ehrenberg, 1833): Arabia, *Panthera pardus saxicolour* (Pocock, 1927): Central Asia, *Panthera pardus melas* (Cuvier, 1809): Java, *Panthera pardus fusca* (Meyer, 1794): Indian sub-continent, *Panthera pardus delacourii* (Pocock, 1930): southeast Asia into southern China, *Panthera pardus japonensis* (Gray, 1862): northern China, *Panthera pardus orientalis* (Schlegel, 1857): Russian Far East, the Korean peninsula and north-eastern China and *Panthera pardus kotiya* (Deraniyagala, 1956): Sri Lanka, this subspecies is endemic to the island [1, 6]. In addition, the leopard populations consist of significant genetic and morphological variation, and in many cases, genetic patterns do not correspond to geographical variation recorded for the particular subspecies [1, 7–11]. Among them, melanistic leopard forms occur throughout their range, mostly in humid and dense areas [12]. Although these initial records suggested the adaptivity for certain ecological conditions, a recent *P*. *p*. *kotiya* melanistic leopard was recorded in Yala National Park. In Sri Lanka, other than the wild type coat coloured leopards, a countable number of melanistic leopards have also been reported from various parts of the island [13]. Apart from Sri Lanka, black leopards have been reported in south-western China, Burma, Assam, and Nepal; from Travancore and other parts of southern India and are also common in Java and the southern part of Malay Peninsula [14]. They are less common in tropical Africa but have been reported from Ethiopia (formerly Abyssinia), the forests of Mount Kenya, and the Aberdares [15, 16]. Sightings have been rare due to their low numbers and their solitary nature. In Sri Lanka, melanistic leopards have reliably been reported from Mavuldeniya, Deniyaya (Sinharaja Rain Forest area) and Nallathanniya highlands, which is a moist densely-forested montane area of the country [13, 17, 18]. Recently a melanistic leopard was spotted in Yala National Park area, in the southern area of Sri Lanka and has been declared a restricted area for visitors (Fig 1) [19, 20] During the last decade in 2009, 2013 and 2020, three melanistic leopards were victims of cruel deaths in poacher’s traps [17, 18, 21]. Some environmentalists claim that the last black leopard in Sri Lanka died in 2020 due to the injuries inflicted by poachers [22].

The density of melanin and the distribution of melanin types on individual hairs determine the coat colour in mammals [23]. Extreme phenotypic changes in the coat colour patterns have been reported in thirteen Felidae species. So far, several melanistic Felidae species have been analyzed [24]. Those exhibit unique mutations associated with two genes; Melanocortin-1 receptor (*MC1R*) [16, 25, 26] and Agouti Signalling Protein (*ASIP*) [16, 26–28]. In leopards, melanism is caused by a recessively inherited mutation in the *ASIP*-gene, which leads to a non-sense mutation ablating *ASIP* function and thus induces black pigmentation [28]. However, in the jaguar, it has been inherited as a dominant trait caused by a 15-base pair deletion in the *MC1R* gene that leads to a gain of function mutation favoring the production of eumelanin [26]. An SNP has been identified at exon 4 of the *ASIP* gene in *Pardofelis temminckii* at position 384 resulting in a non-synonymous substitution associated with black coat colour [28]. Several other complex evolutionary pathways for pigmentation phenotypes in domestic cat [26], puma [26], lynx [29] and ocelot [25, 28] have been studied. Nevertheless, mutations related to melanism in other leopard subspecies are yet to be studied.

Moreover, reconstruction of the phylogenetic history of the leopards provides information on species, subspecies, and population genetic status, which is essential for the conservation of these threatened animals [30]. The Sri Lankan leopard has isolated itself and evolved from dominant intra-guild competition at least since Sri Lanka split off from the Indian subcontinent ~5,000–10,000 ybp [31–33]. It is also the largest land carnivore and apex predator on the island [2, 3, 34, 35]. The Sri Lankan leopard range encompasses a variety of habitats, including the montane, sub-montane, tropical rain, monsoonal evergreen, and arid zone scrub forests [2, 35–37]. Despite its widespread distribution on the island, habitat loss, forest fragmentation, trapping, and hunting pose rising threats to the vulnerable sub-species [33, 38, 39].

Molecular data have strengthened the conservation studies on other cat species; snow leopards, lions, and tigers [40–44], melanism in wolves [45, 46], colour polymorphism in tigers [47] and white-phased black bears [48]. Similarly, genetic analyses provide taxonomic guidelines for subspecies determination in leopards [1]. Previous studies included morphological traits and three molecular genetic methods: allozymes [3], mitochondrial DNA [49] restricted fragment length polymorphism (mtDNA–RFLP), and minisatellites to resolve six phylogeographic groups of leopards, including *P*. *p kotiya* [1]. Intraspecific genetic variation was analyzed using the mitochondrial gene, NADH dehydrogenase subunit 5 (*NADH-5*), which exhibits relatively high rates of mutation in carnivores and has been employed for analysis in the majority of felids, including the leopards [1, 3, 50]. Mitochondrial markers were utilized to identify leopard coalescence and lineage sorting processes [1, 30, 49, 51]. The mtDNA was used to address questions of genetic diversity, phylogeography, and population evolution within Asian leopards, especially the Sri Lankan leopard [52–55].

A broader assessment of its evolutionary history and adaptive significance would assist in better conservation strategies. For example, against this backdrop, the phylogeography of the Sri Lankan melanistic leopard, and how the Sri Lankan leopard, *P*. *p*. *kotiya* emerged are in serious discussion among environmental groups [22, 56].

We utilized data from the melanistic leopard that was killed in 2020, a wild type *P*. *p*. *kotiya*, and published data to assess genetic variation and genetic differentiation in contemporary Sri Lankan leopard populations. We assembled the complete reference genome and *de novo* mitogenome of *P*. *p*. *kotiya* (wild type coat colour) and melanistic coat colour to study its structure and functional components for the subunit 5 of the *NADH* gene, and to compare it with members of the subspecies *P*. *pardus*. Our study aimed to utilize the molecular data, for assessing of the genetic structure of the leopards in Sri Lanka, establish baseline information for future monitoring, and identify genes and mutations for functional melanism.

## Materials and methods

### Ethical clearance

The study was conducted under the research permit WL/3/2/2017/1, issued by the Research Committee of the Department of Wildlife Conservation, Sri Lanka (DWC). We followed relevant guidelines and regulations for animal research approved by the committee. The consent for the study the was taken from the veterinarian surgeon, Department of Wildlife Conservation, Sri Lanka. The DNA samples for sequencing were exported under the Convention on International Trade in Endangered Species of Wild Fauna and Flora (CITES) permit obtained from the DWC.

#### Field observations and morphology analysis

Ten morphometric measurements were taken to the nearest 0.1 cm from a dead black leopard, which died on 29^th^ May 2020 after recovering from a trap at Hatton in Nuwara Eliya district (N- 6.8304983, E- 80.532660). The same animal has been reported in the cameras on 10^th^ December 2019, set up by the DWC. The regular leopard was found at Yala National Park (GPS: N- 6.308977, E- 81.423837. A non-stretch tape was used to measure body, tail, and head to tail length and a graduated wooden sliding calliper was used to record shoulder height and tooth dimensions. A photograph of the right-side profile of each leopard was taken for identification.

#### Sample collection and DNA extraction

About 5 ml of blood was obtained during the post-mortem, from the heart blood and transferred to a tube containing EDTA using a sterile syringe. A similar amount of blood was drawn from a captive wild leopard’s saphenous vein at capture for medical treatment. The DNA was extracted using the Wizard Genomic DNA Purification Kit (Promega, A1120) following the manufacturers’ guidelines and quantified using the Nanodrop 2000 Spectrophotometer (Thermo Scientific). The quality was assessed with Nanodrop readings and running on a 1% agarose gel. Samples were shipped to Admera Health Biopharma Services, New Jersey, USA for sequencing.

#### Genomic library preparation and sequencing

At the sequencing facility, the genomic DNA was quantified with the Qubit 2.0 DNA HS Assay (ThermoFisher, Massachusetts, USA) and assessed for the quality with Tapestation genomic DNA Assay (Agilent Technologies, California, USA). The sequencing libraries were prepared using the KAPA Hyper Prep kit (Roche, Basel, Switzerland) per the manufacturer’s recommendations with Illumina® 8-nt dual indices. The quantity of the final libraries was assessed by Qubit 2.0 (ThermoFisher, Massachusetts, USA), and quality was assessed by TapeStation D1000 ScreenTape (Agilent Technologies Inc., California, USA). Libraries were sequenced on an Illumina HiSeq (Illumina, California, USA) with a read length configuration of 150 PE for 600 M PE reads per sample (300 M in each direction). The preliminary quality assessment of the whole genome sequencing libraries was performed with FastQC v0.11.8 [57]. The FastQC reports were then concatenated using MultiQC v1.9 [58] (36) to create a single diagnostic report containing GC content, sequence quality, duplication level, and adapter content.

#### Reference-based assembly of *P*. *p*. *kotiya* genomes

We used two whole genome sequencing (WGS) datasets for the reference-guided assembly with the chromosome-length *P*. *pardus* genome assembly [59, 60] published by the DNA Zoo team (an improved version of PanPar1.0 [61]) as the reference genome. The reads were mapped to the reference genome with the BWA MEM (v0.7.17) aligner [62], and Samtools’ fixmate [63] was used to correct the aligner’s flaws in read-pairing. Picards’ MarkDuplicates [64] was used to identify duplicate reads in the BAM file, and Samtools’ sort [63] was used to sort the mapped reads in positional order. We used Samtools’ coverage command to generate per scaffold statistics (i.e., mean depth and coverage). Freebayes v1.3.4 [65] was used for variant calling, and variants were filtered by a minimum quality, minimum depth, and maximum depth of 40, 5, and 50, respectively, using VCFtools v0.1.16 [66]. The bcftools (v1.11) [67] stats command was used to generate genotype statistics, and the bcftools consensus command was used to generate the consensus genome sequence.

#### Mutation identification

We selected a set of nuclear genes that code for vital enzymes in melanin synthesis pathways to identify their potential involvement in the formation of its mutations for functional melanism in Sri Lankan black leopard. Therefore, we retrieved Agouti Signaling Protein coding gene (*ASIP)* (Gene ID 109272540), tyrosinase-related protein 1 coding gene (*TYRP1*) (Gene ID 109247101), Dopachrome Tautomerase Protein coding gene (*DCT*) (Gene ID 109264168), OCA2 melanosomal transmembrane protein coding gene (*OCA2*) (Gene ID 109258721), Phospholipase C Beta 4 protein coding gene (*PLCB4*) (Gene ID 109274721), Premelanosome Protein coding gene, (*PMEL*) (Gene ID 109270307), and EGL-9 family hypoxia inducible factor 1 (*EGLN1*) (Gene ID 109248931) gene sequences of *P*. *pardus* from NCBI in order to check which genes affected the phenotype of the black leopards. We mapped the melanocortin 1 receptor gene *MC1R* gene (Gene ID 493917) of *Felis catus* to the *P*. *pardus* reference genome [68] (PanPar1.0 Ensembl version 104) using the Bowtie2 aligner [69] to extract the *MC1R* ortholog of the *P*. *pardus*. The ortholog sequences of these genes were then identified and extracted from the two leopard genome assemblies using the BLASTN function. The reads of the Sri Lankan leopard WGS dataset (ERR5671301) from NCBI were mapped to the coding DNA sequences using the bwa mem aligner [62], and their ortholog consensus sequences were generated using 50% strict calling in Geneious Prime 2020.1.2 software (http://www.geneious.com/). In addition to these sequences, we included the ortholog sequences of the considering genes, which are available in the NCBI. Finally, the multiple sequence alignment of the *ASIP*, *TYRP1*, and *MC1R* orthologs of four *Panthera* samples were executed using the MAFFT v.7.450 [70] with its default configuration. The protein structure of the mutated gene was predicted with RoseTTAFold [71].

#### In-silico analysis for the mutation prediction

The non-synonymous single nucleotide polymorphism (nsSNP) was analysed using four prediction tools: Sorting Intolerant From Tolerant (SIFT) [72], MutPred2 [73], Polymorphism Phenotyping v2 (Polyphen-2) [74], Protein Analysis Through Evolutionary Relationships (PANTHER) [75], and the modelling was performed using SWISS Model [76]. The data for amino acid sequence *ASIP* gene- exon 4, position in the protein, and wild and mutated residue of the nsSNP were used according to the program requirements. The prediction tools were selected by use different approaches in order to obtain a classification of the nsSNP according to one or more features. The tools are freely accessible and described in the literature.

SIFT (https://sift.bii.a-star.edu.sg/) is a sequence homology-based tool with algorithms developed independently. SIFT has a cutoff score of 0.05, and mutations with scores greater than 0.05 are predicted to be damaging [72]. Mutpred2 (http://mutpred.mutdb.org/) is a bioinformatics tool that predicts potential damage and changes in protein properties using sequence homology. A damaging amino acid substitution was defined as one with a general score of >0.5–1 [73]. PolyPhen 2.0 (http://genetics.bwh.harvard.edu/pph2/) is a popular Naive Bayes supervised machine-learning classifier. HumDiv and HumVar were the two algorithms in PolyPhen2. The cut off point was 0.50, and mutations with scores greater than 0.50 were predicted to be damaging [74]. The score of position-specific evolutionary preservations can be calculated by PANTHER-PSEP (http://pantherdb.org/tools/csnpScoreForm.jsp). It calculates the time (in millions of years) that a position in a current protein has been preserved by tracing back to its reconstructed direct ancestors. The longer a position is held, the more likely it will have a negative impact. The thresholds were as follows: "probably damaging" (time > 450my, corresponding to a false positive rate of 0.2 on HumVar), "possibly damaging" (450my > time > 200my, corresponding to a false positive rate of 0.4), and "probably benign" (time 200my) [75].

#### *De novo* assembly and annotation of *P*. *p*. *kotiya* wild type and melanistic leopard

The wild type *P*. *p*. *kotiya* and melanistic leopard mitogenomes were assembled and annotated using the Mitoz v2.3 [77] program. We extracted 5GB of reads from whole genome datasets using the BBMap reformat.sh [78] tool, as recommended by the tool. The generated mitochondrial genomes and annotations were compared with the already available *P*. *pardus* mitogenomes (Table 1). The assembled genomes were visualized with OrganellarGenomeDRAW v1.3.1 [79] software.

**Table 1:** *Pantherinae* and *Felinae* mitochondrial genomes included.

Family	Species	Geographic Location	Source	ID
Pantherinae	*P*. *pardus*	Northern China	EF551002	PPC1
	*P*. *pardus*	Algeria (North Africa)	MH588618	PPA1
	*P*. *pardus*	Cape South Africa	MH588619	PPA2
	*P*. *pardus*	India (Central India)	MH588621	PPF1
	*P*. *pardus*	India (East India)	MH588622	PPF2
	*P*. *pardus japonensis*	Northern China	KJ866876	PPC2
	*P*. *pardus orientalis*	Northern China	KX655614	PPC3
	Wild type *P*. *pardus kotiya*		This study	PPK-W
	Melanistic leopard		This study	PPK-B
	*P*. *leo leo*		KF907306	
	*P*. *leo persica*		NC_018053	
	*P*. *uncia*		EF551004	
	*P*. *onca*		KF483864	
	*P*. *tigris*		EF551003	
	*P*. *tigris altaica*		KF297576	
	*P*. *tigris tigris*		KF892541	
	*P*. *tigris amoyensis*		NC_014770	
Felinae	*Felis catus*		NC_001700	
	*P*. *bengalensis euptilurus*		NC_016189	
	*Acinonyx jubatus*		NC_005212	
	*Puma concolour*		NC_016470	
	*Lynx rufux*		NC_014456	

#### MtDNA divergence dating and nuclear DNA phylogeny

We downloaded 15 Pantherinae mitogenomes and 5 Felinae mitogenomes (Table 1). However, we did not include the partial mitogenomes (MH588618-MH588622) in the divergence dating analysis. The mtDNA D-loop region was excluded from the alignment due to its complexity. We used MAFFT (v.7.450) [80] with its default configuration for multiple sequence alignment. The Bayesian evolutionary analysis was carried out using the BEAST v2.6.5 [81] software package with Markov Chain Monte Carlo (MCMC) procedure to estimate posterior distributions of divergence times and evolutionary dates. The HKY model of evolution and gamma+invariant rate heterogeneity models with a relaxed lognormal clock were used to calculate the substitution rate, while the Yule node calibration technique was used to calculate the divergence. The posterior distributions were calculated using Markov chain Monte Carlo (MCMC) sampling with 100,000,000 steps and 10,000 step logging. We imposed monophyletic constraints for the node to calibrate evolutionary rates, and the times to the most recent common ancestor (TMRCA) of Pantherinae and Felinae with normal priors (10.78 ± 1.87 Mya) [82] were used for the node calibration. Tracer v1.7.2 [83] was used to examine the BEAST output. After discarding 10% MCMC steps as postburn-in, the maximum credibility tree was obtained using TreeAnnotator v2.6.4 (from the BEAST package) and visualized with FigTree v1.4.4 [84].

#### NADH-5 gene phylogeny

The nucleotide variation of the *NADH-5* gene of the Sri Lankan leopard samples was compared to that of the Indian leopards. In addition to *NADH-5* orthologs retrieved from the PPK-W and PPK-B mitogenomes, we included *NADH-5* gene sequences of Sri Lankan and Indian leopards available in the GenBank database (Table 2). A whole-genome sequencing dataset (ERR5671301) of a Sri Lankan leopard was also downloaded from the National Center for Biotechnology Information Sequence Read Archive (NCBI SRA), and reads were mapped to the *NADH-5* sequence (AY035267) of *P*. *pardus kotiya* using the BWA MEM (v0.7.17) aligner [62]. The Geneious Prime 2020.1.2 program was used to generate the consensus sequence, that was included in the analysis. The *NADH-5* orthologs of *P*. *tigris* (EF551003) and *P*. *leo* (KF907306) were identified as the outgroup (Table 1). We used the MEGA X v10.2.4 [70] software to identify the best model of nucleotide substitution based on the Bayesian Information Criterion (BIC). The Kishino and Yano (HKY) + G model of nucleotide substitution (gamma distribution with five rate categories) was identified as the most suitable. The maximum likelihood (ML) tree for *the NADH-5* gene was generated using MEGA X, setting the bootstrap to 1,000 replicates. Additionally, a Bayesian analysis was conducted using Mr Bayes v3.2.6 [85], using the HKG85 substitution model with a chain length of 1,100,000 and 100,000 burn-in. PopArt v1.7 [86] was used to construct a Median Joining haplotype network (ε = 0) of all the sequences from Asian leopards.

**Table 2:** Leopard sequences obtained from Genbank.

	Gene	No. of sequences*	Species	Sample origin	Reference
EF056501.1	*NADH-5*	1	*P*. *pardus*	India	unpublished
AY035270.1-AY035275.1	*NADH-5*	6	*P*. *p*. *fusca*	India	[1]
AY035267.1- AY035269.1	*NADH-5*	3	*P*. *p*. *kotiya*	Sri Lanka	[1]
ERR5671301	Whole genome	1	*P*. *p*. *kotiya*	Sri Lanka	[55]

*The number of sequences for each gene region per species and subspecies utilized in this study.

## Results

### Comparative morphology analysis of regular coat colour and black leopards

**Fig 2:**
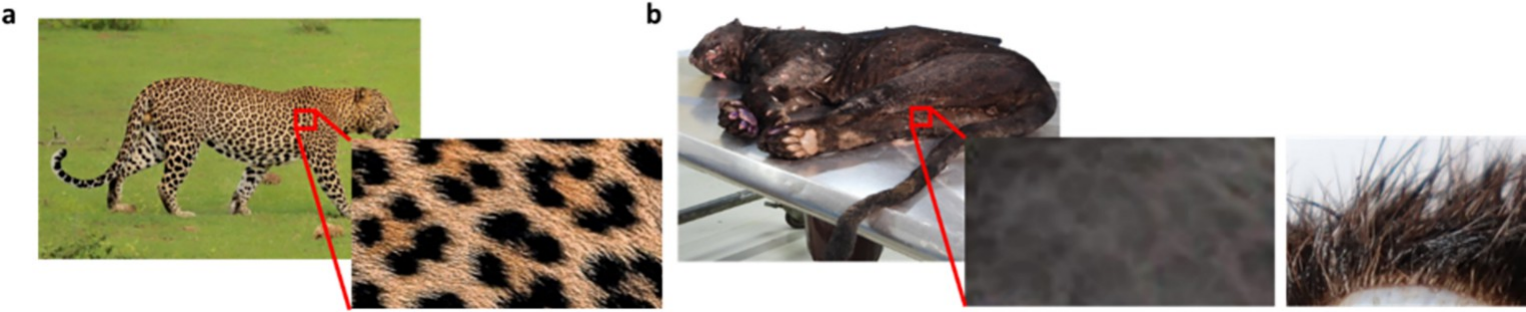
A visual morphological differences between Sri Lankan regular and black leopards. (a) The side view of wild type leopard (PPK-W) and the view of its spot and rosette pattern. (b) The side view of the Sri Lankan melanistic leopard (PPK-B) and the view of its spot and rosette pattern. The spots appear to be still visible in the black background. The cross section of the skin of (PPK-B) with visible fur colouration.

From visual data, the Sri Lankan Leopard’s spot and rosette pattern was distinct from the other leopards [87]. The shapes, sizes, and formations of spot and rosette formations are unique to every leopard [35, 88]. We also compared the spot and rosette formation of animals included in the analysis. The morphological parameters of the two animals included in the analysis were compared (S1 Table). Based on the shreds of evidence, the melanistic leopard is a male animal about ten (10 years old in 2020, while the regular leopard is a female animal about two (2) years old in 2020. The regular coat colour leopard is a striking rust yellow with blackish spots and rosettes. A darker orangish contrast between the spots creates rosettes in some areas of the coat. Rosettes were of different sizes and shapes (Fig 2A). The melanistic leopard also has a distinctive rosette pattern visible even in the darker background (Fig 2B), hidden due to black pigmentation, which was termed “ghost rosettes”.

### Quality checking of the two whole genome sequencing datasets

We generated genome data for two leopard specimens, 100x genome coverage for the melanistic leopard and 30x genome coverage for the wild type coat *P*. *p*. *kotiya*. The obtained WGS datasets consist of 1236.6 and 313 million read pairs for *P*. *p*. *kotiya* and black leopard samples, respectively. These two datasets passed all the FastQC tests except the per sequence GC content test for the reverse reads of the *P*. *p*. *kotiya* library (S1 Fig). Considering the good quality of the sequenced data, we used the original dataset for the downstream analysis.

### Reference guided assembly and identification of *ASIP* mutations

We achieved 90% and 99% of mapping for PPK-B and PPK-W libraries, respectively (Table 3) for paired–end read mapping to the *P*. *pardus* reference genome [59, 60] The mean coverage and mean mapping quality were around 95% and 56, respectively for both samples. As we expected, we have gained mean depths around 100x and 30x. After variant calling, we retrieved only the genotypes with a minimum of 5x depth. Since the sites with unusually high coverage can result in the incorrect mapping of reads from duplicated regions, we set the maximum depth to 50x and 100x for PPK-W and PPK-B, respectively. After the quality filtering step, nearly 55% and 29% of the total genotypes of PPK-B and PPK-W samples were retained (Table 2).

**Table 3:** Statistics of reference-based genome assembly of *P*. *p*. *kotiya* and Black leopard.

	Wild type *P*. *p*. *kotiya* (PPK-B)	Melanisticleopard (PPK-W)
Total read pairs	1,236,550,993	312,991,352
Unmapped reads (%)	264,233,004(10%)	4,085,418 (0.65%)
Duplicates (%)	18.31	7.20
Percentage mean coverage	95.44	95.25
Mean depth	98.79	32.65
Mean mapping quality	55.79	57.41
Number of variant sites	7,977,753	14,939,356
Variant sites after filtering (%)	4,385,747 (54.97%)	43,86,959 (29.37%)
SNPs	3,181,511	3,216,359
MNPs	184,201	162,838
Indels	973,127	957,130

**Fig 3:**
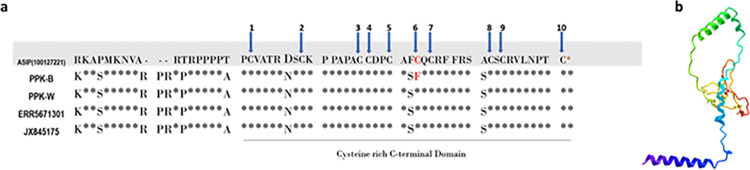
Amino acid alignment of *ASIP*, including the novel *P*. *p*. *kotiya* sequences. (a) Codon Variation in the ***ASIP*** gene Associated with Melanism in the Sri Lankan leopard *P*..*p*. *kotiya*. Amino acid alignment of *ASIP* Exon 4, including the novel **PPK-W** *P*. *p*. *kotiya*- wild and **PPK-B** *P*. *p*. *kotiya-* melanistic. **ERR5671301** wild type *P*. *p*. *kotiya* fossil **JX845175** *Panthera pardus*. Dots indicate the identity of the top sequence; amino acid positions are shown at the end of each line. Dashes represent insertion/deletion (indel) variants. Numbers 1–10 refer to the 10 conserved cysteine residues present in the C-terminal domain. The non-synonymous mutation in melanistic **PPK-B** is indicated in bold and red. (b) Protein structure of the *ASIP* gene in the Sri Lankan melanistic leopard.

For the identification of the cause of melanism, we analyzed the ten genes related to the melanin synthesis pathway as well as genes related to phenotypic variation in the coat. They are Agouti Signaling Protein coding gene (*ASIP*—Gene ID 109272540), Melanocortin-1 receptor (*MC1R* - Gene ID 121942601) tyrosinase-related protein 1 coding gene (*TYRP1*—Gene ID 109247101), Dopachrome Tautomerase Protein coding gene (*DCT*—Gene ID 109264168), *OCA2* melanosomal transmembrane protein coding gene. The *OCA2* (Gene ID 109258721), Phospholipase C Beta 4 protein coding gene *PLCB4* (Gene ID 109274721), Premelanosome Protein coding gene, *PMEL* (Gene ID 109270307), and egl-9 family hypoxia inducible factor 1*EGLN1* (Gene ID 109248931) gene). Out of the analyzed genes, only *ASIP* showed a distinctive variation between the wild type leopards and the melanistic leopards. We aligned our two *ASIP* coding sequences (PPK-W, PPK-B) to those previously available in the leopard data in NCBI. The alignment consisted of 408 bp (136 codons) that exhibited heterogeneous patterns of variation (Fig 3A). The coding region of the *ASIP* gene is highly conserved within the Feliade species, with most of the individuals exhibiting an identical sequence with few exceptions. A Single nucleotide polymorphism (SNP) was identified in exon 4 in the Sri Lankan melanistic leopard. The mutant allele derives a non-synonymous substitution at nucleotide 353 (C353A) predicted to cause a cycteine-phenylalanine substitution at codon 113 (Fig 3B).

### Mutation prediction analysis

The mutation at codon C113F was analyzed to identify whether it affects the protein function. It was damaged by SIFT (score < 0.05) with a score of 0 (Table 4) and predicted to affect protein function. In Polyphen-2, this SNP was detected to be damaging with a score of 0.827 (PSIC > 0.5) (Table 4). The MutPred2 analysis, showed its probability of being a deleterious mutation, with a g score of 0.852 which is greater than 0.5. The program indicated an actionable or confident hypothesis (p score < 0.05) that the molecular mechanism would be disrupted (Table 4). The PANTHER software estimates the likelihood that the nsSNP will affect the function of the protein. The calculated probability of a deleterious (Pdel) effect is 0.57 (Table 4), which is qualitatively predicted as probably damaging. Accordingly, all three tools used suggested that it may be a highly damaging mutation and the MutPred2 tool hypotheses of the molecular mechanism were disturbed, including loss of catalytic residue and loss of stability. The nsSNP C353A showed the highest deleterious scores of the mutations in the SIFT, Polyphen-2, and MutPred2 programs, demonstrating the concordance of the results from the different tools used to predict the most damaging polymorphisms in the *ASIP* gene.

**Table 4:** Prediction scores from Polyphen-2, PANTHER, SIFT and MutPred2 tools of the nsSNP in *ASIP* gene.

Annotation tool	Results
SIFT	0.0
PolyPhen2 PSIC score	0.827
PANTHER Pdel	0.57
MutPred2 g score	0.852
Molecular mechanism disrupted	Altered Metal Binding (p- 0.01)
	Loss of Helix (p- 0.02)
	Loss of disulfide linkage at 119 (p- 0.04)

The GMQE score is 0.12 and QMEAN Z score is below 0.5 (S2 Fig) indicates a model with native quality, as z scores around 0.0 reflect a native-like structure.

### *De novo* mitogenome assembly and divergence dating

**Fig 4:**
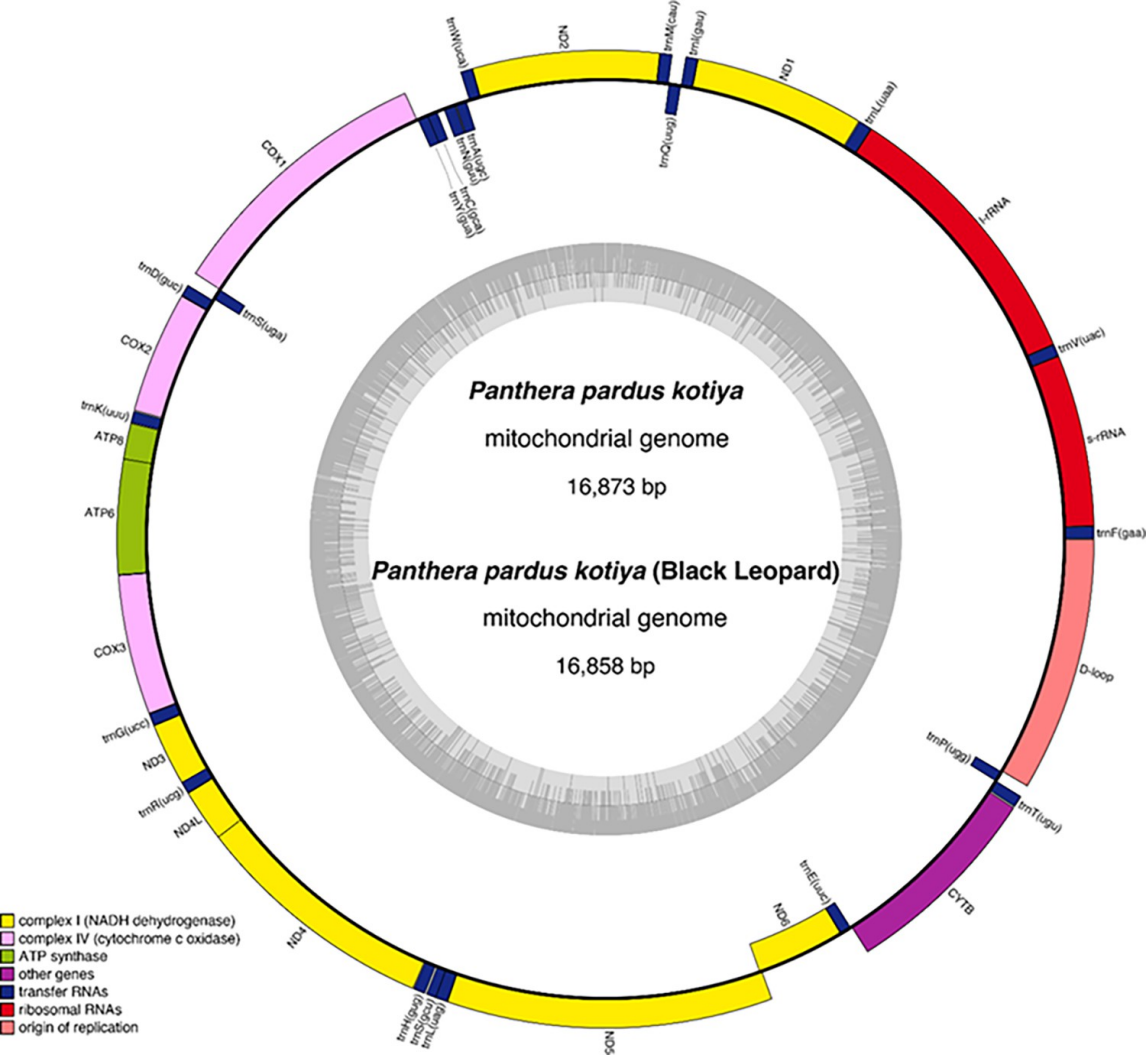
The mitogenome of *Panthera pardus kotiya* and melanistic leopard. Genes on the inside of the circle are transcribed clockwise and those on the outside are transcribed counterclockwise. The different colors represent functional groups, and the darker gray in the inner circle indicates the GC content while the light gray corresponds to the AT content.

We *de novo* assembled the mitochondrial genome of a Sri Lankan leopard using the sequenced WGS dataset, which resulted in a mitogenome of 16,873 bp long (Fig 4) with a 41% GC content. It encodes typical metazoan mtDNA genes, including 13 protein-coding genes (PCGs), 22 tRNA genes, two rRNA genes, and one non-coding control region (D-loop) (S2 Table). The gene order is identical to the *P*. *pardus* (EF551002) mitogenome. The total length of all protein-coding genes in the mitochondrial genome is 11,419bp (i.e., 67.8% of the assembled mitochondrial genome). Similarly, we assembled the mitochondrial genome of the melanistic leopard (Fig 4).

**Fig 5:**
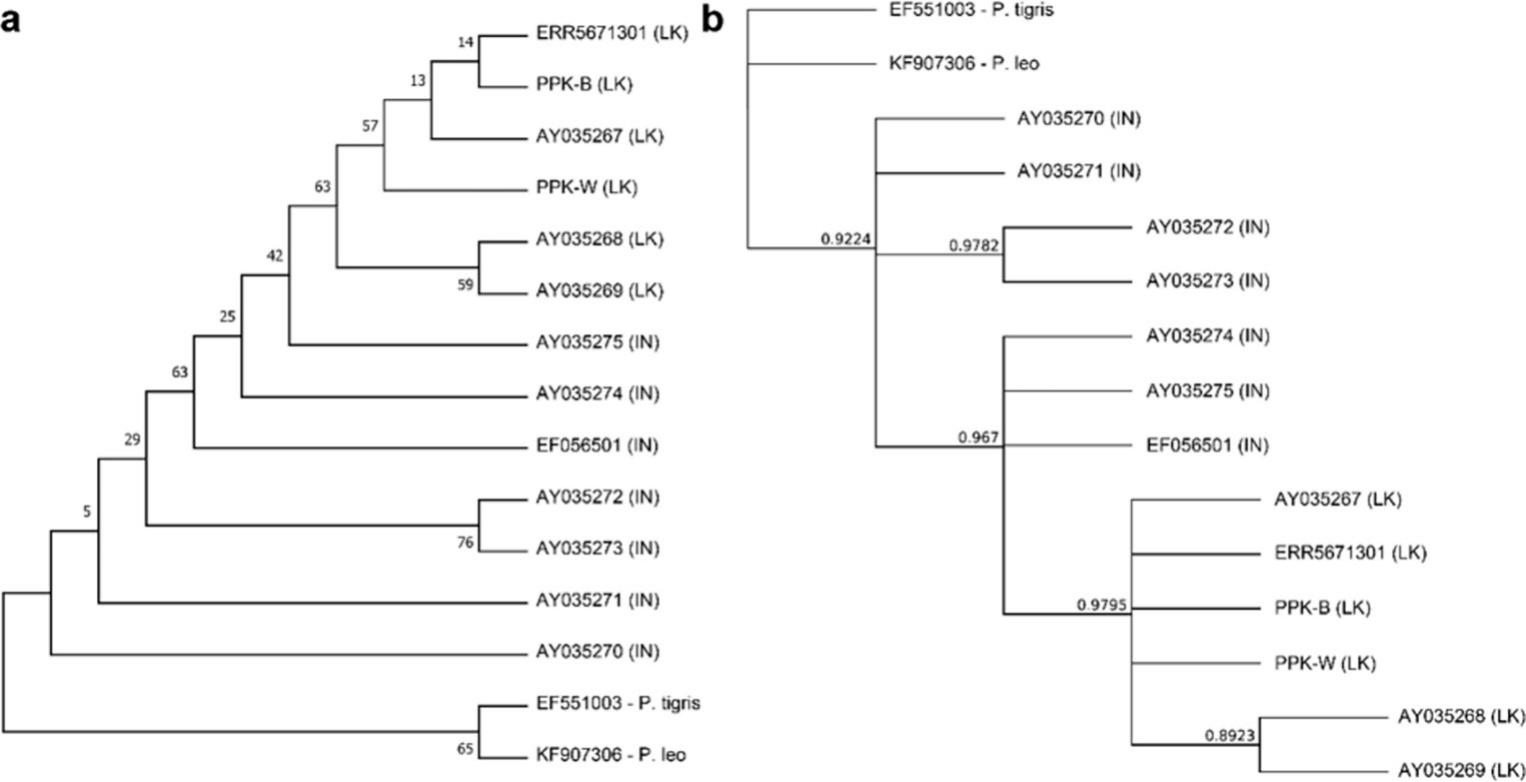
(a) Maximum likelihood (b) Bayesian *NADH-5* gene tree of 13 Asian leopards along with the *P*. *tigris* and *P*. *leo* outgroups. The bootstrap values and posterior probabilities are displayed next to the nodes, respectively. The origins of the samples are given inside the brackets. LK–Sri Lanka, IN—India.

We used *NADH-5* sequences for the downstream analysis because this region has a relatively high rate of mutation in leopards [49]. When the sequences of 7 Indian leopards and 6 Sri Lankan leopards aligned, the ML analysis and Bayesian gene tree grouped the Sri Lankan and Indian leopards into separate groups (Fig 5). Despite the low bootstrap values in the ML tree, it is nearly identical to the Bayesian tree with greater posterior probabilities. Our PPK-W and PPK-B samples were clustered with the Sri Lankan leopard samples. Furthermore, we focused on the links between our samples and the remaining samples in this group. A haplotype network analysis was performed on the same samples to determine the genetic diversity between PPK-W and PPK-B samples and their relationship with the Sri Lankan leopards.

**Fig 6:**
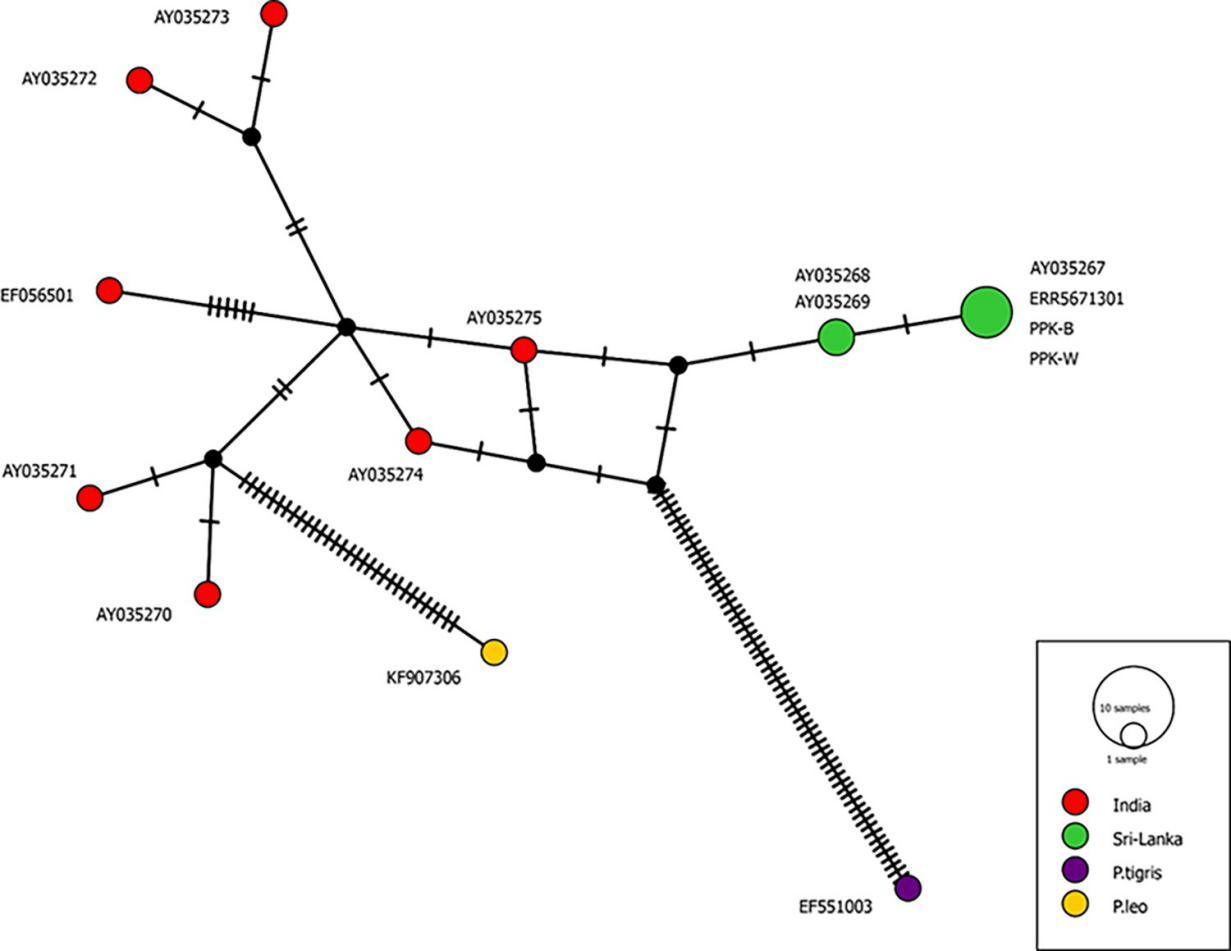
Median joining network analysis of the Asian leopards using *NADH-5* gene. The network includes 6 sequences of Sri Lankan leopards and 7 sequences from Indian leopards. Each node represents a unique haplotype. Haplotypes are colour-coded based on the geographical location/species. Size of the circles represents the number of sequences with the same haplotype. Size of the node reflects the frequency of each haplotype.

In Sri Lankan leopard samples, the sequences provided here identified two unique haplotypes (Fig 6). This haplotype contains our two samples (PPK-W and PPK-B), as well as a previously reported whole-genome sequencing sample (ERR5671301) and is identical to the *Panthera pardus kotiya* haplotype (AY035267.1). Another haplotype was discovered with AY035268.1 and AY035269.1.

**Fig 7:**
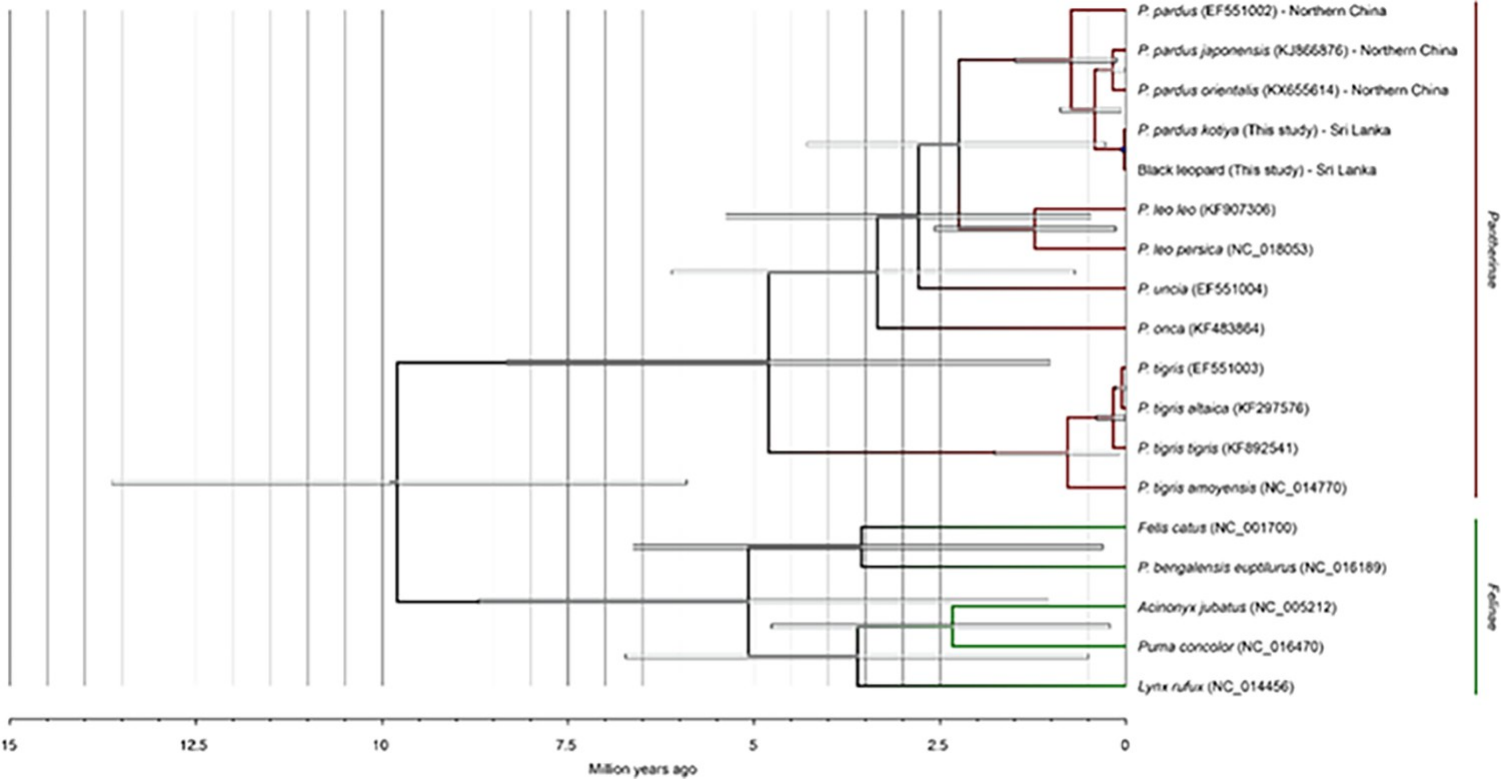
Mitochondrial phylogenetic tree of Pantherinae and Felinae. Node bars represent the 95% highest posterior density (HPD). Tree tips are labeled with the species name, accession number, and origin.

Together with previously published mitogenomes of Pantherinae and Felinae, we derived the phylogenetic relationships and estimated the coalescence times of the considered mitogenomes (Fig 7).

The Bayesian analyses based on 23 complete mitochondrial genomes, excluding the control region, yielded the identical tree topology, supported by a high bootstrap value (above 95%) and high posterior probabilities (above 0.95). The fossil-calibrated Bayesian analysis of the basal divergence time for all Felidae individuals at mitochondrial lineages at 10.78 Mya reconfirms the history. BEAST analysis suggested the divergence time of Felidae individuals was approximately 9.62 Mya from the common ancestor. The divergence of *P*. *tigris* was estimated at approximately 4.9 Mya from the closely related Panthera species (ML, 95%; credibility interval CI 1.5–6.5 My). Divergence of other individuals; *P*. *leo* (lion) at ~2.5 Mya (ML, 95%; CI 0.25–2.75 My), *P*. *uncia* (Snow leopard) at ~2.0 Mya (ML, 95%; CI 0.9–5.5 My). and *P*.*onca* (Jaguar) *~*3.5 Mya (ML, 95%; CI 1.1–6.5 My). The estimation of divergence times of genus Panthera in the present study confirms earlier reports [11, 55, 89, 90]. The Markov Chain Monte Carlo (MCMC) analysis employed in BEAST revealed that *Panthera pardus* split from its sister species leopard at approximately 2.5 Mya. Our results support the deep bifurcation between Asian and African leopards, which has previously been proposed based on short mtDNA sequences [55, 89]. The divergence between the Asian leopards and the East-Central African subspecies is ~1.25 Mya (ML, 95%; CI 0.25–2.5My). The deepest roots within the *P*. *p*. *kotiya* (African origin) were estimated to be ~0.5Mya (ML, 95%; CI 0.25–1.1 My).

## Discussion

The Sri Lankan leopard subspecies *Panthera pardus kotiya* displays a remarkable reduction in endemic genetic diversity, which challenges species conservation in Sri Lanka [91]. A leading cause of the decline of this large carnivore is a conflict with humans, specifically due to livestock depredation. The decrease in habitat and wild prey has increased the number of leopard attacks on domesticated animals. Several leopard deaths have been reported from estate areas [92–94].

Though it is illegal in Sri Lanka, it is not very rare to see snares and traps in the wilderness to kill leopards. However, it is extremely rare to see a trapped melanistic leopard alive and suffering. One such occasion, reported on 20th July 2020, marked the third report of dead melanistic leopards in Sri Lanka. While few citations reports black leopards in Sri Lanka, especially in humid montane forests, no formal studies have been done so far [22, 56]. Our study was based on 100x coverage Illumina data generated from the heart blood of a dead melanistic leopard collected during the postmortem and morphological data collected from the same animal. In addition, we generated 30x coverage Illumina data from a wild type coat colour animal and morphological data from the same and a few others. While we could only include one animal from each phenotype in the molecular study, we assessed both the mitogenome and the nuclear genome.

Initially, to identify the exact genetic interference responsible for melanism, we analyzed the coding sequences of the genes related to the melanin synthesis pathway and receptor genes. In cats, extreme phenotypic changes in the coat colour patterns are associated with single mutations of the *MC1R* and *ASIP* genes [16, 24]. The *MC1R* is a single-exon gene, and mutations change its amino acids, which alter the receptor’s affinity for binding with ligands and G-protein. Eumelanin, the dark pigment, is produced by the *MC1R* gene and activated by the binding of Alpha Melanocyte Stimulating Hormone (α-MSH) [95]. In contrast, the antagonist peptide *ASIP* (Agouti Signaling Protein) binding inhibits MC1R activation, resulting in a switch to pheomelanin (light pigment) synthesis [23, 96]. The *ASIP* gene consists of a peptide of 131 amino acids with a conserved cystein-rich region and a putative signal peptide at the N-terminus [97]. The cysteine-rich domain-containing ten (10) functional cysteine residues, is conserved across taxa [97, 98]. Non-synonymous mutations involving each of the ten cysteine residues of the C-terminal Cys-rich domain, in particular, were found to reduce *ASIP* activity, and its biological activity as an inverse agonist of *MC1R* [27, 99]. The *ASIP* activity was abolished by eight of ten substitutions (at cysteine sites 1–4 and 6–9), while two others (at sites 5 and 10) resulted in partial protein function. Therefore, these cysteine residues are critical for protein activity and receptor binding. The *ASIP* C-terminal loop, a six amino acid segment closed by a final disulfide bond, is essential for high-affinity *MC1R* binding and inhibits the activation of *MC1R* for the production of Eumelanin and leads the production of pheomelanin [100]. Therefore, the gain of function in *MC1R* or the loss of function in *ASIP* induces melanism.

Our results suggest that the SNP identified in the *ASIP* causes melanism in *P*.*p kotiya*. Because of the mutation, the 6th C-residue in the C-terminal loop is substituted by Adenine. This results in a complete loss of its function. When *ASIP* function is completely lost, there will be no effect from this antagonist peptide, and melanistic leopards will have maximum *MC1R* signalling for dark melanin across their entire body. However, more replicated functional studies will be beneficial to further assess the pleiotropic effect in the loci. The four distinct *ASIP* mutations that have been identified thus far to cause melanism in felids appear to result in a total loss of gene function and may have potent pleiotropic consequences. However, there has not yet been any proof of pleiotropic consequences linked to melanism in domestic or wild felids, suggesting that the loss of *ASIP* function solely impacts pigmentation or that it can be made up for in other systems by the activity of other proteins.

In the Sri Lankan leopard, the black rosettes are still visible despite the much-darker background colouration. It is distinct in the cross-section that the beginning of the coated shaft has a cream colouration (Fig 2B). It suggests that it is a mutation of the melanistic coat of the Sri Lankan leopard, and the rosettes are still darker than the darker fur in the background. The functional assays will directly establish the biological effects of these variants. Though several other mutations in the same gene are reported in several other instances, including a nonsense mutation in exon 4 (C333A) and non-synonymous substitution (C384G) and *P*. *pardus* and *P*. *temminckii* respectively, and two more cases in Ocelots [28]. However, the mutation found in the Sri Lankan melanistic leopard, (C353A) has not been reported before. Therefore, this can be considered a novel mutation identified in the *ASIP* responsible for polymorphism in the Felidae family. Further, these non-synonymous mutations in the *ASIP-*gene were evaluated by four software programs that use different methods to predict the damaging nsSNP. All the analyses suggested that the nsSNP damages the regular function of the *ASIP* gene. The results of Polyphen-2, SIFT, MutPred2 and PANTHER analysis, predicted that the resulting mutation is highly damaging, and causes structural changes in the protein.

Previously, it was proposed that leopard colouration possesses adaptive relevance, performing various roles in behavioral and ecological processes [23, 101]. The density of melanin and the distribution of melanin types on individual hairs determine the coat colour of mammals, including leopards [23]. Biological factors such as camouflage, thermoregulation, sexual selection, reproductive success, and parasite resistance could also be the reasons associated with melanism in cats [96, 102]. The occurrence of melanism in leopards has been documented in 13 out of its 37 species [26, 103]. The darker coat colour is considered adaptive to wetter areas with dense vegetation [15, 29]. Even in Sri Lanka, melanistic leopards have mainly been reported in tropical and humid environments. However, in contrast to all these findings, recently a new melanistic leopard was spotted in the Yala National Park, the southern area of the country, which is a tropical region [19] (Fig 1). Therefore it could be assumed that the black colour of the leopard coat could be genetically controlled. However, this phenotype could be advantageous, disadvantageous, or neutral depending on the environment. The evolutionary function of the phenotype could be due to concealment and thermoregulation (evolutionary factor).

The complete mitochondrial DNA sequences provide evidence for molecular dynamics, comparative genetics, and species evolution [104]. We chose mtDNA due to its small genome size, extremely low probability of paternal leakage, higher mutation rate than nuclear DNA, and ability to change mainly through mutation. We *de novo* assembled the mitogenomes of the wild type *P*. *p*. *kotiya* (16,873 bp) and *P*.*p kotiya* melanistic morph, filling a clear gap in understanding the diversity of Sri Lankan leopards since no studies were recorded so far on detailed genome study on *P*. *p*. *kotiya*. Although the divergence analysis highlights the bifurcation between African and Asian leopards and does not confirm the exact origin of *Panthera pardus*, the combined fossil, and genetic evidence together has supported that Africa is the place of origin [1, 6]. All the samples in this analysis clustered within the Asian leopard clade. Our analysis agrees with the previous work that Sri Lankan leopards originated with the migration of the Indian subcontinent [3]. The estimated coalescence time for Sri Lankan leopards is relatively recent.

Our analysis is consistent with that of Miththapala *et al*., in 1991 [105], in which they suggested Sri Lankan leopards have low heterozygosity and polymorphism. Previous studies found low haplotype diversity in leopards who live in West Asian mountains, such as Pakistan, Iran, and Turkmenistan, based on the *NADH-5* gene [5]. Our analysis of the full mitogenomes (excluding the D-loop region) confirmed the low variability in mitogenomic sequences of Sri Lankan leopards, compared to Indian leopards. We excluded the D-loop region because of its high complexity and the occurrence of high repeat regions. The nucleotide variation between the two samples is relatively low, having only six polymorphic nucleotides in the complete mitogenomic region considered. In particular, we selected the *NADH-5* gene previously used in subspecies delimitation of carnivores, showing a higher mutation rate for this group.

However, the haplotype network analysis conducted with current sequences and previous data suggested the presence of two different haplotypes with a single nucleotide variation. The wild type coat colour (BBK-W) and *P*. *p*. *kotiya* black morph (BBK-B) have the same haplotype. The previously recorded sequences, AY035268.1 and AY035269.1 were grouped into separate haplotypes. On the other hand, the *P*. *p*. *kotiya* subspecies may have a low genetic variation, as suggested by Uphyrkina and colleagues grouped the *kotiya* leopard into three haplotypes [1]. This may be due to population isolation and shrinking population sizes due to habitat fragmentation.

Nevertheless, both leopards play a role in ecological overlap within nature and provide suitable conditions for a high level of gene flow between them. In Sri Lanka, melanism is recorded at low frequencies, in contrast to other countries. According to Kingsley [102], *ASIP*-induced melanism is recessive. As a result, it would take a considerable time to rise in frequency under favourable conditions and may linger in the population for a longer period when negatively selected. On the other hand, *ASIP-*induced melanism can reach high frequencies in some populations, suggesting that the trait may be adaptive or at least neutral. Therefore, there is a high probability of the reoccurrence of black leopards in Sri Lanka if the habitat is favourable. One of the main constraints in the conservation of leopards in Sri Lanka is the lack of detailed information, on the leopards and their habitats since those habitats are highly fragmented. Our findings show the importance of cutting-edge technologies for a deep understanding of the germplasm necessary for comprehensive conservation decisions. Including more samples will provide a better resolution and further information for such decision-making strategies.

## Conclusion

Accordingly, Sri Lankan melanistic leopards appears to be a different morph of the Sri Lankan wild type leopards and acquired the colour because of a single point mutation in the ASIP gene. The Sri Lankan melanistic leopards have a different mutation compared to the other recorded *Panther pardus* melanistic leopards. The SNP detected in *Panthera pardus kotiya* causes an amino acid change (C353A) that loss of the function of the *ASIP* gene. Therefore, our study reveals an additional species-specific mutation caused melanism in the Felidae family. As such, the *ASIP* mutation may play an important role in naturally occurring colouration polymorphism. Since this mutation may assist in camouflage, there is a high probability of reoccurrence and inheritance to the next generations. Moreover, the assembled genome could be improved further and annotated using advanced sequencing approaches like HiC and transcriptome assembly.

## Supporting information

S1 Table Genetic contents in the mitochondria genome of *P*. *pardus kotiya*. (DOCX)

S2 Table Morphometric measurements of PPK-W and PPK-B leopards. (DOCX)

S1 Fig GC content test for the reverse reads of *P*. *p*. *kotiya* library. (DOCX)

S2 Fig SWISS modelling analysis for the mutation of C117F. (DOCX)
