# Supplementary material for: A unique single nucleotide polymorphism in Agouti Signalling Protein (ASIP) gene changes coat colour of Sri Lankan leopard (Panthera pardus kotiya) to dark black
Source: PLoS One. 2023 Jul 13;18(7):e0269967. doi: 10.1371/journal.pone.0269967 (PMC10343082; doi:10.1371/journal.pone.0269967)
Supplement: S1 Table — (DOCX) [file pone.0269967.s001.docx]

**Additional File 01:** Genetic contents in the mitochondria genome of *P. pardus kotiya*

| **Functional group** | **Genes** |
| --- | --- |
| Complex I (NADH dehydrogenase subunits) | *nd1,nd2,nd3,nd4,nd4l,nd5,nd6* |
| Complex IV (Cytochrome c oxidase subunits) | *cox1, cox2, cox2* |
| Complex V (ATP synthase subunits) | *atp6, atp8* |
| Cytochrome B | *cytb* |
| Ribosomal RNAs (rRNA) | *12S rRNA, 16S rRNA* |
| Transfer RNAs (tRNA) | *trnA(ugc), trnC(gca), trnD(guc), trnE(uuc), trnF(gaa), trnG(ucc), trnH(gug), trnI(gau), trnK(uuu), trnL(uaa), trnL(uag), trnM(cau), trnN(guu), trnP(ugg), trnQ(uug), trnR(ucg), trnS(gcu), trnS(uga), trnT(ugu), trnV(uac), trnW(uca), trnY(gua)* |
