# Supplementary material for: A unique single nucleotide polymorphism in Agouti Signalling Protein (ASIP) gene changes coat colour of Sri Lankan leopard (Panthera pardus kotiya) to dark black
Source: PLoS One. 2023 Jul 13;18(7):e0269967. doi: 10.1371/journal.pone.0269967 (PMC10343082; doi:10.1371/journal.pone.0269967)
Supplement: S2 Table — (DOCX) [file pone.0269967.s002.docx]

**Additional File 03:** Morphometric measurements of PPK-W and PPK-B leopards

| **Description** | **PPK-W (cm)** | **PPK-B (cm)** |
| --- | --- | --- |
| Estimated total body Length | 230 | 238 |
| Body Length | 98 | 98 |
| Tail Length | 91 | 90 |
| Girth | 93 |  |
| Head Length (upto neck) | 41 | 50 |
| Neck circumference | 58 | 68 |
| Eye-Eye distance |  | 7 |
| Ear-Ear distance | 19 | 16.5 |
| Upper canine - Left | 3.7 | 3.65 |
| Upper canine - Right | 2.6 | 3.8 |
| Lower canine - Left | 3.5 | 2.9 |
| Lower canine - Right | 3.3 | 2.9 |
| Front leg length | 91 | 73 |
| Left front paw - Length | 12 | 15 |
| Left front paw - Width | 10 | 10 |
| Left hind paw - Length | 10 | 9 |
| Left hind paw - Width | 8 | 7 |
