# Supplementary figures and images for: A unique single nucleotide polymorphism in Agouti Signalling Protein (ASIP) gene changes coat colour of Sri Lankan leopard (Panthera pardus kotiya) to dark black
Source: PLoS One. 2023 Jul 13;18(7):e0269967. doi: 10.1371/journal.pone.0269967 (PMC10343082; doi:10.1371/journal.pone.0269967)

**Additional File 02**


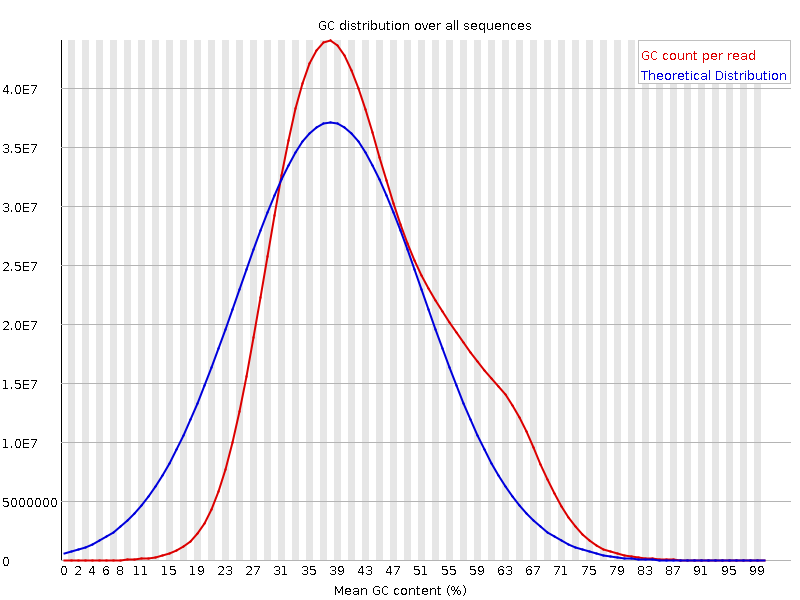

Supplement: S1 Fig — (DOCX) [file pone.0269967.s003.docx]
