# Supplementary material for: A unique single nucleotide polymorphism in Agouti Signalling Protein (ASIP) gene changes coat colour of Sri Lankan leopard (Panthera pardus kotiya) to dark black
Source: PLoS One. 2023 Jul 13;18(7):e0269967. doi: 10.1371/journal.pone.0269967 (PMC10343082; doi:10.1371/journal.pone.0269967)
Supplement: S2 Fig — (DOCX) [file pone.0269967.s004.docx]

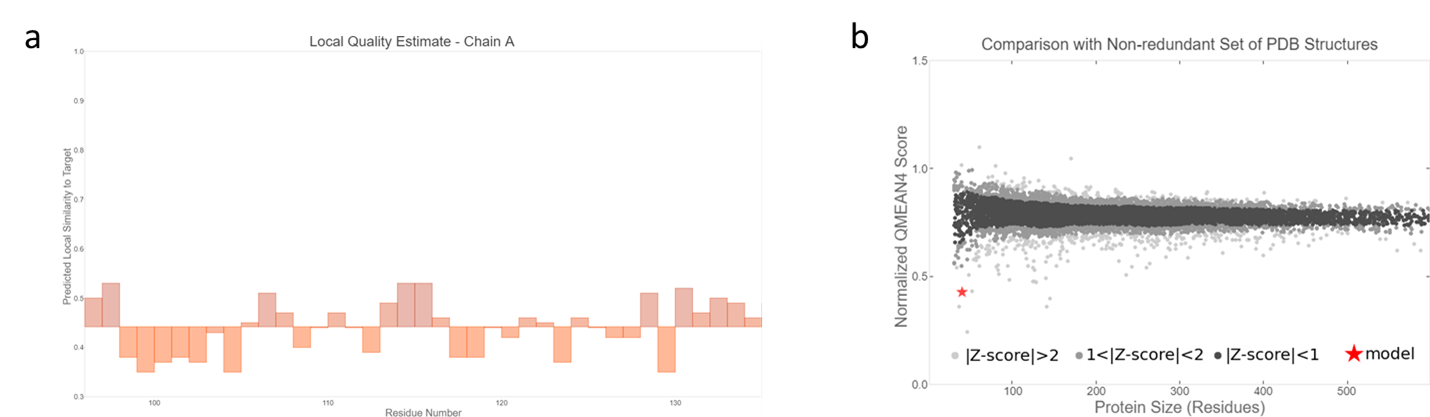


Additional File 3: Figure S2: SWISS Modelling analysis for the mutation of C117F.

1. Local estimates of the model quality based on the QMEAN scoring function and as a global score in relation to a set of high-resolution PDB structures (Z-score).

The score is associated to each residue of the model (reported on the x-axis), reflecting the expected similarity to the native structure (y-axis). Residues show a score below 0.6 which are expected to be of low quality.

1. Quality of the model, as normalised QMEAN score (y-axis), in comparison to the scores obtained for high-resolution crystal structures. The model is less than 0.5 and the quality is comparatively low.
